# Mineral Trioxide Aggregate and Portland Cement for Direct Pulp Capping in Dog: A Histopathological Evaluation

**DOI:** 10.5681/joddd.2014.025

**Published:** 2014-09-17

**Authors:** Maryam Bidar, Neda Naghavi, Nooshin Mohtasham, Mahshid Sheik-Nezami, Amir Fallahrastegar, Farzaneh Afkhami, Negin Attaran Mashhadi, Iman Nargesi

**Affiliations:** ^1^Professor, Department of Endodontics, Dental Research Center, Mashhad University of Medical Sciences, Mashhad, Iran; ^2^Assistant Professor, Department of Endodontics, Dental Research Center, Mashhad University of Medical Sciences, Mashhad, Iran; ^3^Associate Professor, Department of Oral and Maxillofacial Pathology, Oral and Maxillofacial Disease Research Center, Mashhad University of Medical Sciences, Mashhad, Iran; ^4^Endodontist, Private Practice, Mashhad, Iran; ^5^5General Dentist, Private Practice, Mashhad, Iran; ^6^Assistant Professor, Department of Endodontics, Faculty of Dentistry, Tehran University Medical Sciences, Tehran, Iran; ^7^Student of Medicine, Faculty of Medicine, Mashhad University of Medical Sciences, Mashhad, Iran; ^8^Veterinarian, DVM, Private Practice, Mashhad, Iran

**Keywords:** Direct pulp capping, histological evaluation, mineral trioxide aggregate, Portland cement

## Abstract

***Background and aims.*** Mineral trioxide aggregate and calcium hydroxide are considered the gold standard pulp-capping materials. Recently, Portland cement has been introduced with properties similar to those of mineral trioxide aggregate. Histopathological effects of direct pulp capping using mineral trioxide aggregate and Portland cements on dog dental pulp tissue were evaluated in the present study.

***Materials and methods.*** This histopatological study was carried out on 64 dog premolars. First, the pulp was exposed with a sterile bur. Then, the exposed pulp was capped with white or gray mineral trioxide aggregates and white or gray Portland cements in each quadrant and sealed with glass-ionomer. The specimens were evaluated under a light microscope after 6 months. Statistical analysis was carried out using Kruskal-Wallis test. Statistical significance was defined at α=5%.

***Results.*** There was no acute inflammation in any of the specimens. Chronic inflammation in white and gray mineral trioxide aggregates and white and gray Portland cements was reported to be 45.5%, 27.3%, 57.1% and 34.1%, respectively. Although the differences were not statistically significant, severe inflammation was observed mostly adjacent to white mineral trioxide aggregate. The largest extent of increased vascularization (45%) and the least increase in fibrous tissue were observed adjacent to white mineral trioxide aggregate, with no significant differences. In addition, the least calcified tissue formed adjacent to white mineral trioxide aggregate, although the difference was not significant.

***Conclusion.*** The materials used in this study were equally effective as pulp protection materials following direct pulp capping in dog teeth.

## Introduction

In direct pulp capping (DPC) the exposed pulp is covered directly with a biocompatible substance to give the pulp the opportunity to form reparative dentin in order to maintain pulp vitality. This method should be used only on a vital pulp that has been accidentally injured and does not show other symptoms of inflammation. It should not be performed on pulp that has been exposed as a result of penetrating caries. It is particularly important in young adult teeth, where apical root development might be incomplete.^1^

Success rates with direct pulp capping are different depending on the technique and materials. To date, a large number of materials and techniques have been evaluated for direct pulp capping in an attempt to find a reliable bioactive material that stimulates mechanisms of cellular repair, seals the dentin and promotes formation of a stable reparative dentin bridge. However, little success has been achieved. The most famous materials used for this purpose include calcium hydroxide, resins, resin-modified glass-ionomer cements and mineral trioxide aggregate (MTA). In addition, lasers^2^ and bioactive agents such as CEM cement^3,4^ are innovative techniques that stimulate pulpal defense mechanisms.Calcium hydroxide was once considered the standard for pulp capping materials. The major disadvantage of Ca(OH)_2_ is its dissolution over time. It may induce reparative dentin formation, but most dentin bridges formed under Ca(OH)_2_ may contain tunnel defects which allow the pulp to become infected or necrotic over time.^5^

MTA is a powder containing fine hydrophilic particles; it has recently attracted attention for pulp capping treatment because of its small particle size, its excellent sealing ability,^6^ its biocompatibility,^7^ alkaline pH value when set, its ability to stimulate hard tissue formation^8^ and antimicrobial effects.^9^

The difference between two forms of MTA (gray MTA and white MTA) has been reported to be in the concentrations of aluminum, magnesium, and iron compounds. White MTA lacks the aluminoferrite phase that imparts the gray color.^10^ Several studies have shown no significant differences between GMTA and WMTA in terms of biocompatibility and cell response.^11,12^

MTA and Portland cement (PC) seem almost identical macroscopically, in microscopic views and by x-ray diffraction analysis.^13^ There have been no differences in the biological effects of MTA and Portland cement in several studies. However, the two substances are not exactly the same:^14,15^ MTA contains a smaller quantity of gypsum than PC, is composed of smaller particles, and incorporates bismuth oxide to improve radiopacity.^15^ Portland cement still needs to be tested extensively before it can be used in clinical dentistry.

There are only a few published studies on animal teeth that investigate the interaction of PC with the pulp of permanent teeth. The study described in this paper was designed to evaluate the inflammation, presence of necrosis, formation of hard tissue, evaluation of fibrous tissue and status of blood vessels in the pulp of dog teeth to two forms of MTA, gray Portland cement and white Portland cement when used as direct pulp capping materials.

## Materials and Methods

In this animal study sixty-four premolars from 4 male adult mixed-breed dogs were used for the purpose of histopathological evaluation; the animals were about one year old. The apexes of premolars in dogs become closed at 10th month of age. We needed teeth with closed apexes for this study. The 1-year-old dogs were used for homogeneity of the samples.

The guidelines for animal ethics in Iran were followed. The study protocol was approved and conducted in accordance with the policies and principles of laboratory animal care of Mashhad University of Medical Sciences in Iran. Each animal was anesthetized with an intramuscular injection of 10 mL of ketamine; lidocaine (Persocaine, Daroopakhsh, Iran) was used for local anesthesia. Then each quadrant was isolated by a rubber dam and the occlusal cavity was prepared close to the pulp by using a carbide fissure bur in a high-speed handpiece, with sterile saline as the coolant. The pulp of each tooth was exposed by trephination with a #2 round carbide bur (Tizkavan, Tehran, Iran). Bleeding was controlled by sterile cotton pellets. The exposed pulp was irrigated with 2.5% NaOCl and capped with one of the following materials in each quadrant: (1) ProRoot gray MTA (ProRoot; Dentsply Tulsa Dental, OK, USA); (2) ProRoot white MTA (Dentsply Tulsa Dental, OK, USA); (3) gray Portland cement (Mashad Cement Co., Iran); (4) white Portland cement (Mashad Cement Co., Iran). Gray and white Portland cements were sterilized by ethylene oxide. All the materials were mixed in a 3:1 powder:distilled water ratio.

A wet cotton pellet was placed over the materials for complete setting and the teeth were covered by Cavit (3M ESPE, Seefeld, Germany) for the next 48 hours. After that, the animals were anesthetized again, the cotton pellets were removed and the teeth were covered by a resin-modified glass ionomer cement (Vitrabond; 3M ESPE, St Paul, MN) and light-cured for 40 seconds for sealing. All the animals were then injected with 24 mL of 10% dextrose serum (Daroo-pakhsh Co., Iran) subcutaneously, to prevent side effects from general anesthesia during recovery, such as mortality/morbidity due to lack of appetite and glucosuria.

After six months, the animals were euthanized with an anesthetic overdose. Vital perfusion with 10% formalin through the common carotid artery (using Pedrello pump, made in Italy) was performed.^16^ Both jaws were removed, fixed in a 10% neutral-buffered formalin solution, and decalcified in Surgipath Decalcifier I (Surgipath, Grayslake, IL, USA). Tissue blocks were dehydrated and embedded in paraffin. Six-micron histological slices were prepared in a buccolingual direction from the samples, stained with hematoxylin & eosin, and histologically studied under a light microscope (Leika Gallen III microscope, Model BME, 13395H2X, Buffalo, NY, USA) using ×40 and ×100 magnifications. The sections were examined by a pathologist blinded to the source of the specimens. Every sample was evaluated for pulp inflammation, pulp necrosis, formation of a dentin bridge, formation of fibrous tissue and vascularity in the vicinity of the test materials.

To assess inflammation, the inflammatory cell counts were determined in 100 μm^2^ of the high-density area. According to the number of the cells, the specimens were classified in the following groups based on a study by Ørstavik and Mjör:^17^

- without inflammation: 0−1 cell
- mild inflammation: 2−5 cells
- severe inflammation: >15 cells

If there were lymphocytes, plasma cells or macrophages in this area, it was classified as chronic inflammation and if there were PMNs, it was classified as acute inflammation.

Necrosis was defined as destruction and pyknosis of nucleoli, in addition to eosinophilic cytoplasm or loss of cellular shape.

To determine the amount of fibrous tissue, if collagen fibers could be observed in the form of short fibers in one-third of the pulp chamber, it was reported as low and if they were observed in more than one-third of the pulp chamber it was reported as high.

The scores for vascularity were: normal, low and high. To determine vascularity, if the number of blood vessels counted in 5 HPF (high-power field) was less than 10, blood vessel changes were reported as low and if it was more than 10, blood vessel changes were reported as high.

Non-parametric Kruskal-Wallis test was used for statistical analysis. Statistical significance was defined at α=5%.

## Results

A total of nine samples from gray MTA, and white and gray PC were eliminated because of some drawbacks in preparation techniques. The results were divided into five sections as follows:

### Status of Inflammation Based on Acute and Chronic Inflammation

PMNs were not observed in the specimens, indicating that none of the groups had acute inflammation, but chronic inflammatory cells were reported in 45.5%, 27.3%, 57.1% and 34.1% of white and gray mineral trioxide aggregates and white and gray Portland cements, respectively. There were no significant differences between the study groups in terms of chronic inflammation (P=0.212; Table 1).

**Table 1:** Status of chronic inflammation and severity of chronic inflammation in study groups

	White MTA	Gray MTA	White PC	Gray PC	P-value
Chronic inflammation	45.5%	27.3%	57.1%	34.1%	P=0.212
Severe inflammation	18.2%	9.1%	14.3%	13.2%	P=0.29

**Figure 1.**
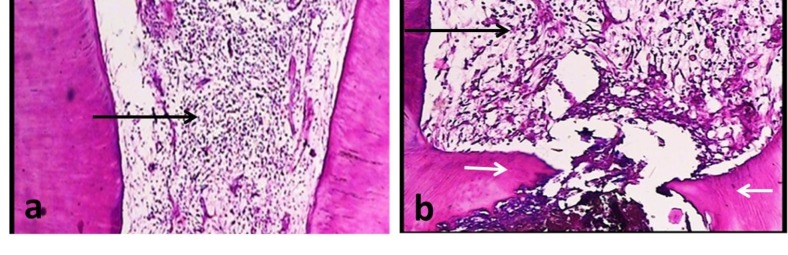
Histological images of inflammatory cell infiltration (H&E staining technique). (a) Severe chronic inflam-mation (black arrow) in the vicinity of gray MTA, original magnification, ×40. (b) Severe chronic inflammation (black arrow) and calcified tissue formed (white arrows) in the vicinity of white MTA,original magnification, ×100.

Severe inflammation (more than 15 inflammatory cells in 100 μm^2^ of the high-density area) was highest in the white MTA group, although not significant (P = 0.29; Table 1; Figure 1).

### Pulp Status Based on the Presence of Necrosis

Of all the samples, 13 samples were completely necrotic. White MTA exhibited the highest rate of necrosis, with white Portland cement exhibiting the lowest rate of necrosis. However, the differences between the groups were not significant (P=0.206).

### Formation of Hard Tissue

**Figure 2.**
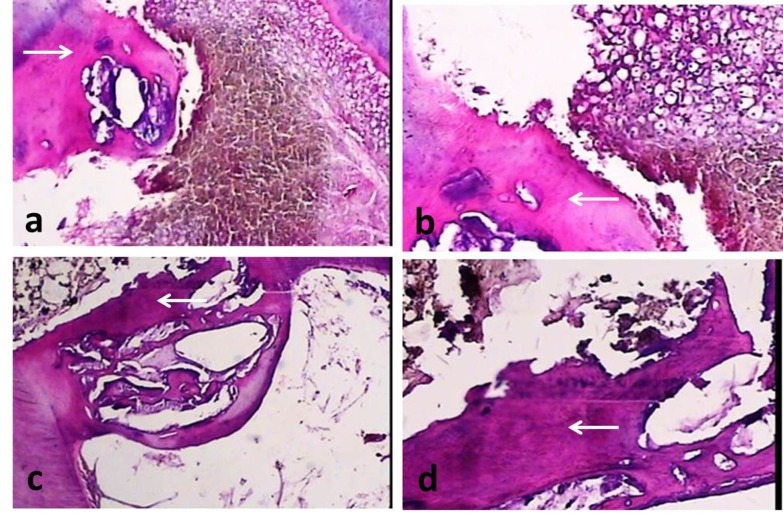
Histological images of calcified tissue formation (H&E staining technique). (a) Osteoid formation (white arrow) in the vicinity of white PC, original magnification, ×40. (b) Osteoid formation (white arrow) in the vicinity of gray PC, original magnification, ×100. (c) Dentinoid formation (white arrow) in the vicinity of white MTA, original magnification, ×40. (d) Dentinoid formation (white arrow) in the vicinity of gray MTA, original magnification, ×100.

The hard tissues formed in the samples were dentinoid, osteoid or amorphous in light microscopic view. White Portland cement exhibited the highest rate of hard tissue formation (85.7%); however, the differences between the groups were not significant (P=0.22; Figure 2).

### Evaluation of Fibrous Tissue

White MTA group had the lowest rate of fibrous tissue; in 72.7% of the samples in this group no fibrous tissue was observed. Data analysis showed no significant differences between the groups (P=0.458).

### Status of Blood Vessels in the Region

Pulp status based on the vascularity was normal in 45.5%, 41.7% 57.8% and 64.4% of white and gray mineral trioxide aggregate and white and gray Portland cement samples, respectively. White MTA resulted in the highest rate of blood vessel formation (more than 10 blood vessels in 5 HPF), but the differences were not significant (P=0.25).

## Discussion

In recent decades researchers have made great efforts to discover materials that, in addition to being used for direct capping of vital pulp tissue, prepare a base for its healing. DPC is a method in which an exposed pulpal tissue is directly covered by a substance to give the pulp the opportunity to form restorative dentin and maintain pulp vitality.^1^

This study was performed on upper and lower premolar teeth of dogs because of their similarity in size and length of roots to human molar teeth. In addition, dogs are strong animals that are able to open their mouth well, making it easy to work on their teeth. According to Ford et al,^18^ a dog’s anatomy is suitable to make it an experimental model.

Portland cement has been used as an apical plug material, for perforation repair, as root end filling material, for pulp capping and pulpotomy in several studies.^19-21^In the present study histopathological effects of direct pulp capping were evaluated using mineral trioxide aggregate and Portland cements on dental pulp tissue. Consistent with previous studies, in this study the application of white MTA and gray MTA, and white and gray Portland cement materials on pulp as capping materials did not result in significant differences in terms of inflammation, pulp necrosis, formation of hard and fibrous tissue and increased vascularity.^13,22^

There was no acute inflammation in any of the specimens; however, chronic inflammation was observed in 45.5% of the white MTA specimens, 27.3% of the gray MTA specimens, 57.1% of the white Portland cement specimens, and 34.1% of the gray Portland cement specimens, which might relate to the high surface pH, especially when these materials are freshly mixed, causing denaturation of adjacent cells and tissue proteins.^13^ As the materials set, the pH changes and the cell injuries subside.

Among the groups with chronic inflammation, the white MTA group exhibited the highest percentage of severe inflammation, the highest rate of blood vessel increase, and the lowest increase in fibrous tissue. Data analysis showed no significant differences between the groups.

In a similar study Menezes et al evaluated the histological effects of these four materials (gray and white MTA, gray and white PC) on dog teeth (incisors, canines and premolars) when used as a wound dressing in pulpotomy. They reported that the tissue reaction patterns and cell reactions were identical for ProRoot MTA, MTA Angelus, Portland cement and white Portland cement. They attributed the ability of MTA and Portland cement to support the formation of a dentin bridge to high sealing ability and fast setting. This prevents diffusion of the material into surrounding tissues and reduces microleakage during the healing period.^13^ They emphasized that such pulpotomy procedures should be carried out under ideal conditions, i.e. all the teeth should be free of caries and pulp inflammation, which might impair the healing process. In the present study intact teeth were used, which were free of pulp inflammation.

It is known that the presence of bacteria is a significant inhibiting factor for healing of pulp exposures. In the present study, no sections were stained for bacterial presence. The Portland cements were sterilized by ethylene oxide to avoid bacterial contamination and yet preserve characteristics of the materials. This might not have been necessary owing to the high pH of the materials, in which microorganisms are less likely to survive.

Menezes et al^13^ reported no necrosis in their specimens, but there were 13 samples with complete necrosis in the present study, which might be attributed to the longer experimental period in this study (6 months versus 4 months) and the differences in methods (pulp capping versus pulpotomy and premolar teeth versus incisor, canine and premolar teeth). Considering the differences in the results of these two studies it would be acceptable that pulpotomy is a more successful treatment than pulp capping with these four materials. In another study the process of dental pulp repair following pulpotomy and pulp capping with these materials was compared. In all the samples, a complete hard tissue bridge was formed and both materials yielded similar results.^22^

Some authors have reported that MTA and Portland cement release calcium ions when they are in contact with tissue fluid, thus promoting an alkaline pH. This hydration produces calcium silicate hydrate gel and calcium hydrate, which would explain why MTA and Portland cement provoke the same tissue reaction.^23^

In a study by Dreger etl,^24^ it was concluded that MTA was more effective in promoting the biomineralization process than Portland cements.

In 2003, Saidon et al^14^ implanted MTA and Portland cement in the mandible of an Indian guinea pig and evaluated the cellular and tissue reactions of these two materials. The results indicated that biocompatibility properties of these materials were comparable, and Portland cement can be used as a cheaper alternative for MTA.^14^ The same results were achieved in the study of Kurita et al^25^ with ProRoot MTA, Portland cement and MTA Bio in mice connective tissue; however, Shahi et al^26^ concluded that MTAs (gray and white) were more biocompatible than gray and white Portland cements.

Recently several new compositions of Portland cement such as EPC (a mixture of epoxy resin and Portland cement)^21^and TheraCal (resin and Portland cement)^27^have been introduced and compared with MTA in some properties. The results offer major advantages for them.

The PC comes in numerous varieties depending on the geographical source; some contains arsenic and possibly other dangerous contaminants. This material still needs to be tested extensively before it can be used in dentistry, and therefore no recommendation can be made for human use. Further studies should be carried out and limitations and the potential unknown risks involved in the use of Portland cement as a medical device should be considered to ensure its safety.

## Conclusion

The results of the present histological study showed that, in non-carious dog teeth, there appear to be no differences between MTAs (gray and white) and PCs (gray and white) in terms of inflammation, pulp necrosis, formation of hard and fibrous tissue and increased vascularity. Nevertheless, these results were obtained in healthy pulp tissue and the correlation with the response in inflamed pulp should be made with caution. In addition, further research with larger samples and a more extended study time is necessary. Clinical studies should also be encouraged.

##  Acknowledgments

The present study was part of a student thesis (number: 2177). Authors acknowledge the financial support for this research project by the Research Council of Mashhad University of Medical Sciences. The authors deny any conflict of interests.

## References

[1] Dammaschke T (2008). The history of direct pulp capping. J Hist Dent.

[2] Moritz A, Schoop U, Goharkhay K, Sperr W (1998). The CO2 laser as an aid in direct pulp capping. J Endod.

[3] Fallahinejad Ghajari M, Asgharian Jeddi T, Iri S, Asgary S (2010). Direct pulp-capping with calcium enriched mixture in primary molar teeth: a randomized clinical trial. Iran Endod J.

[4] Asgary S, Nosrat A, Homayounfar N (2012). Periapical healing after direct pulp capping with calcium-enriched mixture cement: a case report. Oper Dent.

[5] Cox CF, Sübay RK, Ostro E, Suzuki S, Suzuki SH (1996). Tunnel defects in dentin bridges: their formation following direct pulp capping. Oper Dent.

[6] Torabinejad M, Waston TF, Pitt Ford TR (1993). Sealing ability of mineral trioxide aggregate when used as a root end filling material. J Endod.

[7] Masuda YM, Wang X, Hossain M, Unno A, Jayawardena JA, Saito K et (2005). Evaluation of biocompatibility of mineral trioxide aggregate with an improved rabbit ear chamber. J Oral Rehabil.

[8] Koh ET, Torabinejad M, Pitt Ford TR, Brady K, McDonald F (1997). Mineral trioxide aggregate stimulates a biological response in human osteoblasts. J Biomed Mater Res.

[9] Saatchi M, Hosseini HS, Farhad AR, Narimany T (2012). The effect of various concentrations of iodine potassium iodide on the antimicrobial properties of mineral trioxide aggregate–a pilot study. Dental Traumatology.

[10] Asgary S, Parirokh M, Eghbal MJ, Brink F (2005). Chemical differences between white and gray mineral trioxide aggregate. J Endod.

[11] Bidar M, Zarrabi MH, Afshari JT, Aghasizadeh N, Naghavi N, Forghani MR (2011). Osteoblastic cytokine response to gray and white mineral trioxide aggregate. Iran Endod J.

[12] Camilleri J, Montesin FE, Papaioannou S, McDonald F, Pitt Ford TR (2004). Biocompatibility of two commercial forms of mineral trioxide aggregate. Int Endod J.

[13] Menezes R, Bramante CM, Letra A, Carvalho VG, Garcia RB (2004). Histologic evaluation of pulpotomies in dog using two types of mineral trioxide aggregate and regular and white Portland cements as wound dressings. Oral Surg Oral Med Oral Pathol Oral Radiol Endod.

[14] Saidon J, He J, Zhu Q, Safavi K, Spangberg LS (2003). Cell and tissue reactions to mineral trioxide aggregate and Portland cement. Oral Surg Oral Med Oral Pathol Oral Radiol Endod.

[15] Dammaschke T, Gerth HU, Zuchner H, Schafer E (2005). Chemical and physical surface and bulk material characterization of white ProRoot MTA and two Portland cements. Dent Mater.

[16] Sabeti MA, Nekofar M, Motahhary P, Ghandi M, Simon JH (2006). Healing of apical periodontitis after endodontic treatment with and without obturation in dogs. J Endod.

[17] Orstavik D, Mjör IA (1992). Usage test of four endodontic sealers in Macaca fascicularis monkeys. Oral Surg Oral Med Oral Pathol.

[18] Ford TR, Torabinejad M, Abedi HR, Bakland LK, Kariyawasam SP (1996). Using mineral trioxide aggregate as a pulp-capping material. J Am Dent Assoc.

[19] Chakraborty A, Dey B, Dhar R, Sardar P (2012). Healing of apical rarefaction of three nonvital open apex anterior teeth using a white portland cement apical plug. Contemp Clin Dent.

[20] Silva Neto JD, Schnaider TB, Gragnani A, Paiva AP, Novo NF, Ferreira LM (2012). Portland cement with additives in the repair of furcation perforations in dogs. Acta Cir Bras.

[21] Lee SJ, Chung J, Na HS, Park EJ, Jeon HJ, Kim HC (2013). Clin Oral InvestigCharacteristics of novel root-end filling material using epoxy resin and Portland cement. Clin Oral Investig.

[22] Holland R, de Souza V, Murata SS, Nery MJ, Bernabé PF, Otoboni Filho JA (2001). Healing process of dog dental pulp after pulpotomy and pulp covering with mineral trioxide aggregate or Portland cement. Braz Dent J.

[23] Holland R, de Souza V, Nery MJ, Otoboni Filho JA, Bernabé PF, Dezan Júnior E (1999). Reaction of rat connective tissue to implanted dentin tubes filled with mineral trioxide aggregate or calcium hydroxide. J Endod.

[24] Dreger LA, Felippe WT, Reyes-Carmona JF, Felippe GS, Bortoluzzi EA, Felippe MC (2012). Mineral trioxide aggregate and Portland cement promote biomineralization in vivo. J Endod.

[25] Kurita LM, Cavalcante RB, Gurgel-Filho ED, De-Deus GA, Ximenes AB, Da Silva EJ (2013). Response of mice connective tissue to three different endodontic materials. Microsc Res Tech.

[26] Shahi S, Rahimi S, Yavari HR, Mokhtari H, Roshangar L, Abasi MM (2010). Effect of mineral trioxide aggregates and Portland cements on inflammatory cells. J Endod.

[27] Gandolfi MG, Siboni F, Prati C (2012). Chemical-physical properties of TheraCal, a novel light-curable MTA-like material for pulp capping. Int Endod J.

